# Effects of Various LED Light Spectra on Growth, Gonadal Development, and Growth-/Reproduction-Related Hormones in the Juvenile Red Spotted Grouper, *Epinephelus akaara*

**DOI:** 10.3390/ani13132047

**Published:** 2023-06-21

**Authors:** Wengang Xu, Huafeng Zou, Jun Zeng, Weiping Mei, SongHee Choi

**Affiliations:** 1 School of Ocean, Yantai University, Yantai 264003, China; 2 Key Laboratory of Exploration and Utilization of Aquatic Genetic Resources, Ministry of Education, Shanghai Ocean University, Shanghai 201306, China; hfzou@shou.edu.cn; 3 Guangxi Academy of Sciences, Nanning 530007, China; junzeng@gxas.cn (J.Z.);; 4 Institute of Beibu Gulf Marine Industry, Fangchenggang 538000, China; 5 Marine Science Institute, Jeju National University, Jeju 63333, Republic of Korea; thdgml1126@naver.com

**Keywords:** brain–pituitary axis, growth hormone, pre-pro-somatostatin-I, neuropeptide Y, cholecystokinin, kisspeptins, G protein-coupled receptor 54, gonadotropin-releasing hormone, gonadotropins

## Abstract

**Simple Summary:**

Most studies have focused on water temperature and photoperiods on the effects of growth and reproduction in fish, but the light spectrum also plays an important role in inducing the synthesis and secretion of growth-/reproduction-related hormones. In this study, the juvenile red spotted grouper was exposed to four light spectra from white, red, blue, or green light-emitting diodes (LEDs) for two months. The fish exposed to white LEDs exhibited the best growth, although the expression levels of growth hormone (GH) showed no significant differences and the pre-pro-somatostatin-I (PSS-I) expression levels significantly increased. Four LED spectra were effective in stimulating food intake as the expression levels of neuropeptide Y significantly increased. The gonadal development advanced from chromatin nucleolar-stage oocytes to perinucleolar-stage oocytes in all four LED groups, while there were no significant differences in FSHβ and LHβ expression. This result suggests that FSHβ and LHβ may not have played important roles in gonadal development when these fish were exposed to the four light spectra. Collectively, our results provide evidence that the natural light condition (white LED condition) is a better choice for the growth of juvenile red spotted grouper in captivity during summer.

**Abstract:**

The light spectrum is a key environmental cue involved in growth and reproduction in teleosts. This study investigated the effects of exposure on juvenile red spotted grouper exposed to white (control), red (590 nm), blue (480 nm), and green (520 nm) light-emitting diodes (LEDs) (12 h light:12 h dark) for two months. The body weight (BW), total length (TL), condition factor (CF), weight gain rate (WGR), gonadosomatic index (GSI), and hepatosomatic index (HSI) were assessed. Gonadal development was observed. The gene expression of growth-related hormones, such as growth hormone (GH), pre-pro-somatostatin-I (PSS-I), neuropeptide Y (NPY), and CCK, and of reproduction-related hormones, such as Kiss1, Kiss2, GPR54, sbGnRH, FSHβ, and LHβ, was analyzed. The results showed that the fish in the white LED group exhibited the best BW, TL, CF, WGR, and HSI after one or two months. The fish exposed to white LEDs showed the best growth after two months, but no significant differences in GH levels were detected. Contrarily, the expression levels of the PSS-I significantly increased (*p* < 0.05) in fish from the white group, suggesting the complex regulation of GH production and the limited effects of PSS-I on the inhibition of GH synthesis and somatic growth. The significantly increased NPY levels in the four LED groups (*p* < 0.05) indicated that these four LED spectra were effective in stimulating food intake and energy homeostasis. After two months, the gonads developed from chromatin nucleolar-stage oocytes to perinucleolar-stage oocytes in the four LED groups. The gene expression of Kiss2 and GPR54 in the four LED groups and of sbGnRH in the white and blue LED groups significantly increased when compared to that in the initial group (*p* < 0.05), while there were no significant differences in FSHβ and LHβ expression in the four LED groups. These results suggest that FSH and LH may not play important roles in gonadal development in juvenile red spotted grouper that are exposed to these four LED spectra.

## 1. Introduction

The red spotted grouper, *Epinephelus akaara*, is widely distributed in China, Japan, Korea, and other Asian countries and is recognized as one of the most important marine fish due to its high-quality meat and good commercial value [1]. Recently, most studies focused on the effects of environmental factors, such as water temperature and photoperiods [2,3], on the growth and development of this species. However, there are also reports that light spectra can influence the entire life cycle of teleost fish, from embryonic development to sexual maturation [4,5,6,7]. For example, the best performance of larvae was achieved under light conditions in the European sea bass, *Dicentrarchus labrax* [8], and the red spectrum was able to reduce the growth of juvenile gilthead seabream, *Sparus aurata* [9]. Furthermore, light spectra are also considered to be effective for the induction of gonadal maturation in reef fish [10]. For example, three species of coral reef fish—Apogon compressus, *Apogonidae*, Pomacentrus amboinensis, *Pomacentridae*, and Premnas biaculeatus, *Pomacentridae*—exhibited spectral sensitivity in a variable-light environment during the larval and juvenile stages [11]. It is widely known that the red spotted grouper lives in a colorful environment and appears to be very sensitive to the color of light, as it is a coral reef fish [12]. However, until now, there is little information on the effects of light spectra on growth and gonadal development in this species.

In teleosts, the brain-pituitary (BP) axis is considered the master axis of the endocrine system, and it regulates many functions, such as growth, reproduction, metabolism, energy homeostasis, and so on. Light spectra may change circadian rhythms, regulate the secretion of some hormones of the BP axis, and, thus, affect growth and gonadal development. Growth hormone (GH) is synthesized and released mainly in the pituitary gland, and it functions as an important regulator of metabolism and somatic growth [13]. Pre-pro-somatostatin-I (PSS-I), the precursor gene of somatostatin (SS), is synthesized in the brain and is a major inhibitor of basal and stimulated GH secretion [13]. Brain neuropeptide Y (NPY) and cholecystokinin (CCK) are hormones produced by neuroendocrine and digestive endocrine cells, and they are involved in the intake regulation and feeding behavior of fish [14]. It has been reported that NPY stimulates food consumption, affects blood pressure, induces anxiolysis, enhances memory retention, and affects circadian rhythmicity [15]. CCK acts in the hypothalamus, where it confers satiety, and in the gastrointestinal tract, where it promotes the secretion of pancreas enzymes, peristalsis, and gallbladder contraction [14,16,17]. Generally, these hormones have strong interactions and cooperate to regulate somatic growth and energy homeostasis in teleosts. In female goldfish, NPY-induced GH release was blocked by SS; however, an intraperitoneal injection of NPY induced time- and dose-dependent increases in serum GH levels [18]. Light spectra affected growth via the regulation of the GH and PSS-I levels. Shin, Lee, and Choi [19] reported that compared to red light, green and blue lights were more effective in enhancing GH levels and accelerating growth in yellowtail clownfish, *Amphiprion clarkii*.

The effects of light spectra on maturation and reproduction are directly achieved by altering the secretion of gonadotropins (GtHs) in the pituitary gland, which has been reported in the European perch, *Perca fluviatilis* [20], and grass puffer, *Takifugu niphobles* [21]. Two types of GtHs, follicle-stimulating hormone (FSH) and luteinizing hormone (LH), are regulated by gonadotropin-releasing hormone (GnRH) in the hypothalamus [22]. Recently, it was identified that there is upstream control by kisspeptin to regulate the secretion of GtHs. Brain kisspeptin, which includes the products of the Kiss1 and Kiss2 genes [23], binds to its G-protein-coupled receptor 54 (GPR54) and directly innervates the GnRH neurons [24]. Shin, Habibi, and Choi [25] and Choi et al. [26] reported the effects of light spectra on the reproduction-related hormones in the BP axis in goldfish, *Carassius auratus*. In our recent study, we showed that light spectra had great effects on growth in tiger puffer, *T. rubripes* [27], and on maturation in grass puffer [21]. Therefore, this study aimed to determine the effects of various light spectra on growth and gonadal development in juvenile red spotted grouper. Furthermore, the gene expression of the *GH*, *PSS-I*, *NPY*, and *CCK* growth-related hormones, as well as that of the *Kiss1*, *Kiss2*, *GPR54*, *sbGnRH*, *FSHβ*, and *LHβ* reproduction-related hormones in the BP axis, was examined in this species.

## 2. Materials and Methods

### 2.1. Experimental Fish and Conditions

**Figure 1:**
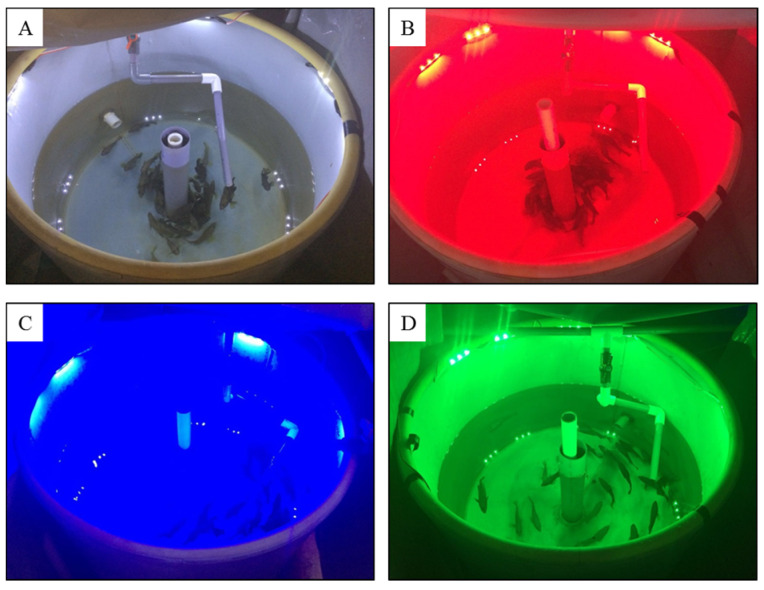
The experimental tank of fish exposed to white (**A**), red (**B**), blue (**C**), and green (**D**) light-emitting diodes.

One-year-old red spotted grouper (n = 160; body weight, 42.29 ± 0.77 g; total length, 15.6 ± 0.07 cm) were purchased from CR Co., Ltd., Jeju, Republic of Korea, and were allowed to acclimate for one week in four 500-L tanks equipped with re-circulation filter systems and aeration in the laboratory. Each tank (each experimental group) contained 40 fish and was covered on the sides with black plastic sheeting to avoid sunlight. The fish were exposed to white (control), red (590 nm), blue (480 nm), or green (520 nm) light-emitting diodes (LEDs, SS Light Co., Ltd., Seoul, Republic of Korea) for two months from 24 July to 23 September 2018. Eight 0.72 W LEDs with a light intensity of about 580 lux were placed 22–25 cm above the water surface, and the irradiance at the water surface was maintained at approximately 14 W/m^2^ (Figure 1).

**Figure 2:**
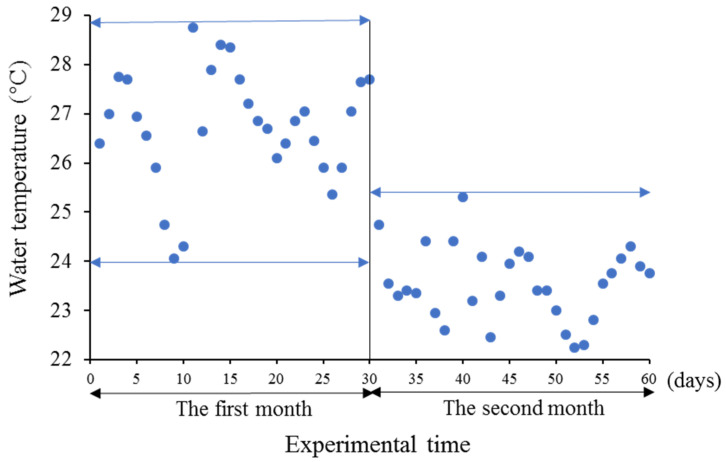
Changes in water temperature during the duration of the experiment from 24 July to 23 September 2018. The data are from the database of the Korea Meteorological Administration.

The water temperature was the natural seawater temperature and ranged between 22.3 and 28.8 °C (Figure 2), and a 12 h light (L):12 h dark (D) period (with a digital timer; lights on at 06:00 h and lights off at 18:00 h) was implemented. In the first month, the water temperature ranged from 24.1 to 28.8 °C. In the second month, the water temperature ranged from 22.3 to 25.3 °C. The fish were fed with commercial pellets (Eureka Co., Ltd., EP4, Busan, Republic of Korea) twice daily (10:00 h and 18:00 h), and no fish died during the experimental period.

### 2.2. Calculations of Growth Parameters

The body weight (BW) and total length (TL) of each fish in each tank were measured at the beginning of the experiment and after one and two months of light exposure. The gonadal weight (GW) and liver weight (LW) of the fish were randomly selected and measured before the beginning of the experiment, and each fish was measured after two months of light exposure.

Other growth parameters, including the condition factor (CF), weight gain rate (WGR), specific growth rate (SGR), gonadosomatic index (GSI), and hepatosomatic index (HSI), were calculated as follows:

CF = 100 × (BW/TL^3^) (1)

WGR (%) = 100 × [(FW − IW)/IW] (2)

SGR (%/day) = 100 × [(Ln FW − Ln IW)/T] (3)

GSI (%) = 100 × (GW/BW)(4)

HSI (%) = 100 × (LW/BW)(5)

FW and IW denote the final mean weight and initial mean weight, respectively. T represents the rearing days.

### 2.3. Sampling Procedures and Sample Preparation

Fish were anesthetized with 2-phenoxyethanol (Junsei Chemical Co., Ltd., Tokyo, Japan). The dosages of anesthesia for various teleosts [28] were used as base information, and the concentration was 400 µL/L. Before the start of the light experiment, 8 fish were sacrificed for the initial sampling. After two months, 8 fish in each light group were sampled. The gonads were extracted and fixed in Bouin’s solution. The brains and pituitary glands were collected and stored at −80 °C until analysis. All of the tissues in Bouin’s solution were fixed for 24 h and stored in 70% ethanol until histological processing.

### 2.4. Histological Procedures on Gonads

After being fixed in 70% ethanol, the gonads were embedded in paraffin wax and cross-sectioned by using a microtome (RM2125 RTS, Leica Biosystems, Heidelberger, Germany) at a thickness of 5 or 7 μm. The sections were dehydrated with gradient ethanol and stained with hematoxylin-eosin. Histological sections were examined with an optical microscope and digitally photographed (Olympus BX53, Tokyo, Japan).

### 2.5. Total RNA Extraction and cDNA Synthesis

Total RNA was extracted from the brain and pituitary gland of each fish by using RiboEx LS^TM^ reagent (GeneAll Biotechnology Co., Ltd., Seoul, Republic of Korea) and quantified with a NanoVue spectrophotometer (GE Healthcare Life Sciences, Buckinghamshire, UK). For cDNA synthesis, the total RNA from the tissue was treated with DNase by using an RQ1 RNase-Free DNase kit (Promega Corporation, Madison, WI, USA) and reverse-transcribed by using PrimeScript^TM^ 1st-strand cDNA synthesis (Takara Bio., Inc., Otsu, Shiga, Japan). In detail, 1 μg of RNA from the brain or 200 ng from the pituitary gland was diluted with RNase-free water to a total of 8 µL in the PCR tube. This mixture was reacted with 1 µL of random hexamer primer (50 µM) and 1 µL of dNTP mixture (10 mM each), followed by incubation at 65 °C for 5 min. Subsequently, 10 µL of this mixture was added with 4 µL of 5X PrimeScript Buffer, 0.5 µL of RNase Inhibitor (40 U/µL), 1 µL of PrimeScript RT (200 U/µL), and 4.5 µL of RNase-free water. A total of 20 µL of mixture was incubated at 30 °C for 10 min, 42 °C for 60 min, and 95 °C for 5 min.

### 2.6. Quantitative Real-Time PCR

The mRNA expression of *PSS-I*, *NPY*, *CCK*, *Kiss1*, *Kiss2*, *GPR54*, and *sbGnRH* in the brain and of *GH*, *FSHβ*, and *LHβ* in the pituitary was examined. The primers of each gene were designed according to the GenBank accession number and are listed in Table 1. QPCR reactions were performed on a CFX96 Touch^TM^ Real-Time PCR System (BioRad, Hercules, CA, USA) by using EvaGreen 2X qPCR MasterMix-Rox (Abm Inc., Richmond, BC, Canada) under the following thermal cycle: initial denaturation at 95 °C for 10 min, then 40 cycles that included 94 °C for 45 s, 58 °C for 45 s, and 72 °C for 1 min. At the end of the PCR reaction, melting curve analyses were performed, and the *β-actin* gene was used as an internal control for the relative quantification. Each 10 µL of QPCR reaction contained 2 µL of cDNA, 5 µL of EvaGreen 2X qPCR MasterMix (Abm Inc., Richmond, British Columbia, Canada), 0.3 µL of each primer (10 pM), and 2.4 µL of RNase-free water.

### 2.7. Statistical Analysis

All data were analyzed by using the SPSS 23.0 software (IBM, Armonk, NY, USA) and were expressed as means ± SEM. The differences were tested by using a one-way analysis of variance (ANOVA), followed by Tukey’s Honestly Significant Difference (HSD) test (*p* < 0.05).

## 3. Results

### 3.1. Growth Performance

After one month of maintenance under different LED spectra, the BW and CF in the fish in the white group were significantly higher than those in other groups (*p* < 0.05). The TL of the fish in the white group was also significantly higher than that of the fish in the blue and green groups (*p* < 0.05) (Table 2). After two months of maintenance under different LED spectra, the BW of the fish in the white group was significantly higher than that of those in the green group (*p* < 0.05). The fish in the white group also exhibited a significantly higher TL than that of those in the blue and green groups (*p* < 0.05). Furthermore, the WGR of the fish in the white group was higher than that of those in the other groups. The HSI of the fish in the white group was significantly higher than that of those in the initial groups (*p* < 0.05). However, the fish in the green group exhibited the lowest BW, TL, CF, and WGR (Table 2).

### 3.2. Gonadal Development

**Figure 3:**
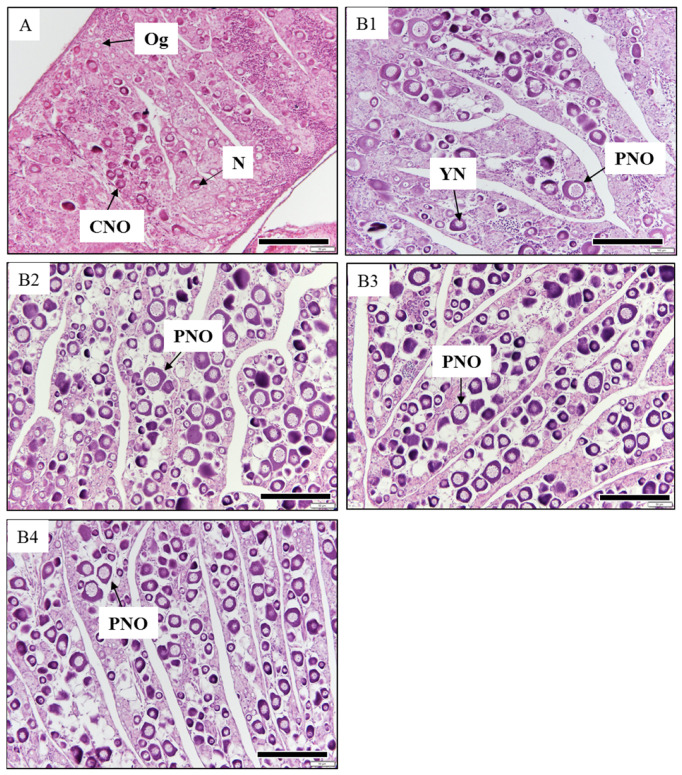
Gonadal development of the red spotted grouper *Epinephelus akaara* in the initial group and in fish in the four LED groups after two months of LED exposure. (**A**) initial group; (**B1**) white LED group; (**B2**) red LED group; (**B3**) blue LED group; (**B4**) green LED group. Og, oogonia; CNO, chromatin nucleolar-stage oocyte; N, nucleus; PNO, perinucleolar-stage oocyte; YN, yolk nucleus. Scale bar = 200 μm.

At the beginning of the experiment (initial group), the gonads were in the chromatin nucleolar stage of oocyte development (Figure 3A). Some chromatin nucleolar-stage oocytes were observed in the ovary, which appeared slightly larger than the oogonia. The oocytes had a thin cytoplasm and a large nucleus occupying the greater part of the oocyte. After two months, the gonadal development of the fish in all four LED groups had advanced to the perinucleolar stage, with oocytes exhibiting the presence of strong basophilic cytoplasm (Figure 3(B1–B4)). After two months, a yolk nucleus appeared and increased in size, and a few nucleoli were distributed around the inner margin of the nuclear membrane. The cytoplasm of the oocytes greatly increased in volume.

### 3.3. Expression of Growth-Related and Reproduction-Related Genes

**Figure 4:**
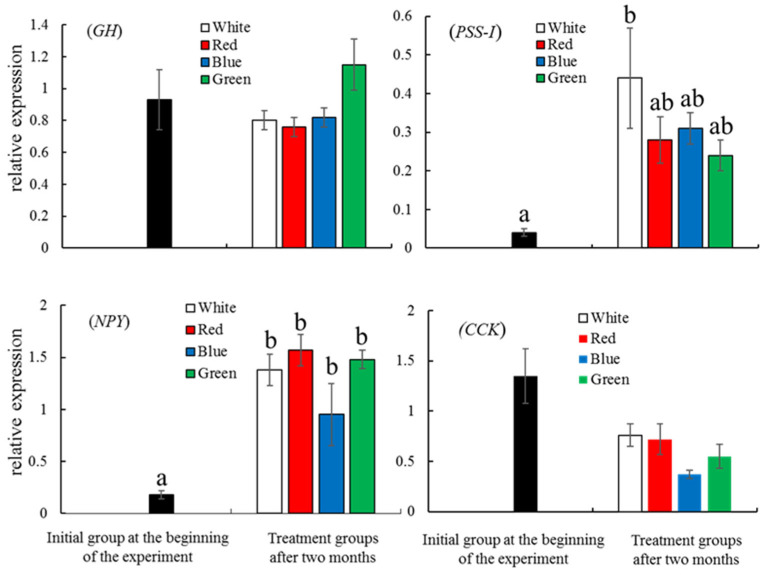
The normalized expression of the growth-related genes *GH, PSS-I*, *NPY*, and *CCK* in red spotted grouper *Epinephelus akaara* in the initial group and in the fish in the four LED groups after two months of LED exposure. All data are shown as means ± SEM. Data with different letters are significantly different (*p* < 0.05).

**Figure 5:**
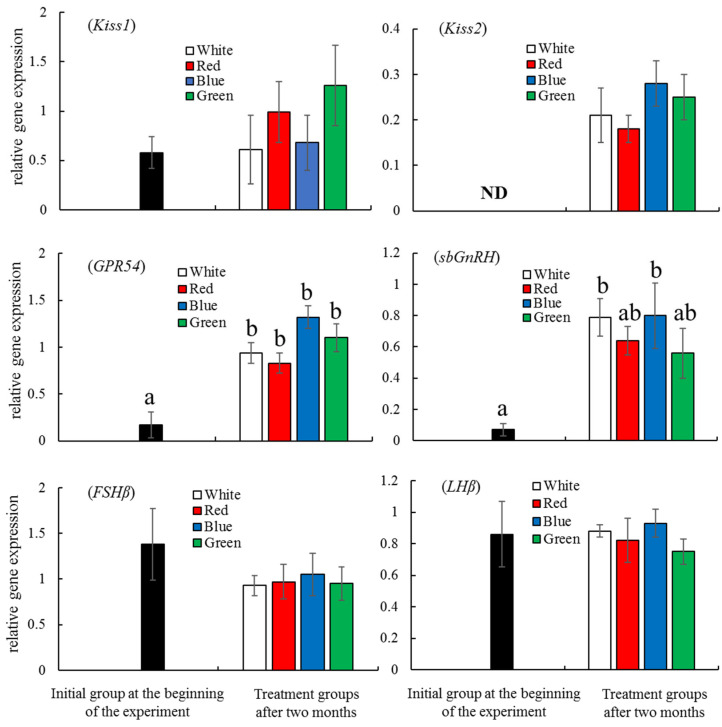
The normalized expression of the reproduction-related genes *Kiss1*, *Kiss2*, *GPR54*, *sbGnRH*, *FSHβ,* and *LHβ* in red spotted grouper *Epinephelus akaara* in the initial group and in the fish in the four LED groups after two months of LED exposure. All data are shown as means ± SEM. ND, non-detectable. Data with different letters are significantly different (*p* < 0.05).

As shown in Figure 4, the normalized expression levels of *PSS-I* in the fish in the white LED group and the *NPY* levels in the fish in all four LED groups were significantly higher than those in the initial group (*p* < 0.05), while the *GH* and *CCK* expression levels showed no significant differences among the LED groups. After two months of LED treatment, there were no significant differences in the expression of *Kiss1*, *FSHβ*, and *LHβ* between the initial and LED treatment groups, but the *Kiss2*, *GPR54*, and *sbGnRH* expression in the fish in the four LED groups shower higher levels than those of the fish in the initial group (Figure 5). The expression level of *Kiss2* in the initial group was undetectable. The expression levels of *GPR54* in the fish in the four LED groups were significantly higher than that in the initial group (*p* < 0.05). The expression levels of *sbGnRH* in the white and blue LED groups were significantly higher than that in the initial group (*p* < 0.05) (Figure 5).

## 4. Discussion

In this study, the red spotted grouper that was exposed to white LEDs for two months exhibited the best BW, TL, WGR, and HSI in comparison with those in the other LED groups. The slowest growth was exhibited by fish exposed to green LEDs. However, in our previous studies, when the water temperatures ranged from 13.6 to 16.7 °C, juvenile red spotted grouper exposed to blue light for ten weeks exhibited the best BW [29]. Furthermore, the highest growth rates were observed under green light in longtooth grouper, *E. bruneus* [30], and the lowest mortalities were observed with red light in orange-spotted grouper, *E. coioides* [31]. These different results indicate that when grouper species are exposed to similar light conditions, the environmental factors, such as water temperature, experimental time, and feeding conditions, may have effects on growth. Different results also appeared in other species. In Nile tilapia, *Oreochromis niloticus*, Luchiari and Freire [32] found that red light might have some harmful effects on the growth rate, but Volpato et al. [33] reported that red light stimulated feeding. However, there was also a report that blue light achieved better BW and TL in Nile tilapia [34]. Furthermore, rainbow trout, *Oncorhynchus mykiss*, showed an a greater TL when reared under red light than under white and blue lights [35], while it was also reported that blue light sources may be suggested for fish welfare in cultures of this species [36]. Barfin flounder, *Verasper moseri*, reared under green or blue light exhibited a greater BW and TL than those under red light [37]. These results show that light spectra may be a limited factor of fish growth, and the response of growth to light spectra may vary depending on the rearing conditions, size, age, season, maturity, and so on in teleosts.

In this study, the CF in the white LED group was significantly higher after one month, but there were no significant differences after two months. In the first month, from 24 July to 23 August, the average water temperature was 26.7 °C, which is the optimal temperature for the growth of red spotted grouper, as it is a type of warm-water fish [38]. However, in the second month, from 24 August to 23 September, the average water temperature was only 23.4 °C. In previous studies, it was shown that higher water temperatures could induce faster growth of channel catfish, *Ictalurus punctatus* [39], largemouth bass, *Micropterus salmoides* [40], and red spotted grouper [41]. In this study, it was possibly the higher water temperature and white LED that caused the red spotted grouper to achieve a significantly higher CF in the first month.

After two months, expression levels of *NPY* significantly increased in all four light groups in comparison with the initial group, suggesting that these four light spectra are effective in inducing food intake, metabolism, and energy homeostasis. In this study, although the fish exposed to white LEDs exerted the best somatic growth after two months, the *GH* expression levels in the white group showed no significant differences when compared to those in the other LED groups. The ability of exogenous GH to promote and increase the growth rate has been widely reported in salmonids [42,43], but there is little doubt that endogenous GH is a principal regulator of somatic growth [44]. Generally, GH secretion is activated when GH-releasing hormone (GHRH) binds to GH receptors (GHRs), and these factors stimulate the secretion of insulin-like growth factor-I (IGF-I) in the liver, which, in turn, directly regulates growth. Interestingly, although the best growth appeared to be in the fish in the white LED group in this study, the expression levels of *PSS-I* in the white group significantly increased after two months. PSS-I exists as a type of precursor of SS, which inhibits GH secretion in all vertebrate species studied [45]. In this study, the *PSS-I* and *GH* levels between the white and green LED groups indicated contrary trends, suggesting that PSS-I inhibited GH secretion. Ben-Shlomo and Melmed [46] reported that although SS is an important inhibitor of GHRH action, the peptide plays a less prominent role in maintaining baseline GH secretion from the pituitary gland. In this study, although the *PSS-I* level in the white LED group significantly increased, it may not have greatly inhibited GH secretion, resulting in only limited inhibition of growth. However, it was shown that green and blue light enhanced the GH levels in yellowtail clownfish [19]. The growth of fish is widely influenced by many external factors, as well as endocrine factors, such as cortisol, thyroid hormones, reproductive hormones, and ghrelin. Together, these factors create a complex regulation of GH production [47]. Therefore, to avoid the interference of hormones from other tissues, further study should be focused on in vitro culture of brain or pituitary cells exposed to various light spectra to understand the regulation of GH.

After two months, the stage of oocyte development advanced from the chromatin nucleolar stage during the initial sampling to the perinucleolar stage in all four LED groups. Over this time period, the gene expression of *Kiss2* and *GPR54* in all four light groups and of *sbGnRH* in white and blue groups also significantly increased. However, there were no significant differences in *FSHβ* and *LHβ* expression among the four light groups. Recently, the endocrine system of the Kiss-GnRH-GtHs axis was considered a potent regulator of reproduction in grass puffer [48], European sea bass [49,50], striped bass, *Morone saxatilis* [51], and red spotted grouper [41]. Until now, FSHβ has been widely known to be active as a key hormone in mediating early vitellogenesis and spermatogenesis, whereas LHβ is generally associated with final maturation and ovulation or spermiation in the salmonid fish [52,53,54], Japanese conger, *Conger myriaster* [55], Japanese eel, *Anguilla japonica* [56], and European sea bass [57]. All of these results indicate that the roles and functions of FSHβ and LHβ are closely related to the puberty and maturation phases in teleosts. Tanaka et al. [58] reported that in the artificial environment of a fish farm, red spotted grouper begins puberty at two years old and matures at three years old. In this study, the fish were only one year old, and the gonadal development stage was that of primary oocyte development, which was far from puberty. Therefore, even if the *Kiss2*, *GPR54*, and *sbGnRH* levels significantly increased, they may not have effectively induced the synthesis and secretion of *FSHβ* and *LHβ* in this phase, which would result in no significant differences in the expression of the two hormones’ beta subunits. A similar finding was observed in European perch, where Brüning et al. [20] reported that when male and female perch were exposed to the control, red, green, or blue light for 10 days, no significant differences were found in the expression of *FSHβ* and *LHβ*, as the reproductive cycle had not yet begun in this fish. However, significant increases in *FSHβ* and *LHβ* were observed in goldfish that were exposed to green light due to the presence of oocytes in the yolk stage [25]. Therefore, in a further study, we should choose pubertal or adult red spotted grouper to investigate the effects of different LED spectra on the reproductive system.

## 5. Conclusions

**Figure 6:**
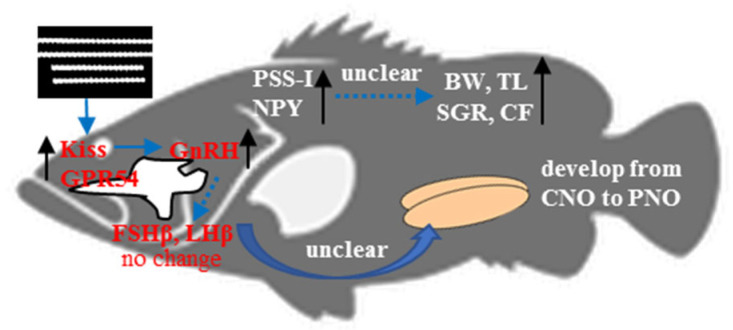
The effects of white LEDs on the growth, gonadal development, and growth-/reproduction-related hormones in red spotted grouper *Epinephelus akaara*.

In summary, as shown in Figure 6, the juvenile red spotted grouper exposed to white LEDs exhibited the best growth after two months. However, in the white group, the expression levels of *GH* showed no significant differences. In contrast, the expression levels of *PSS-I* significantly increased, suggesting the complex regulation of GH production and the limited effects of PSS-I on somatic growth. Significant increases in the expression levels of *NPY* in all four LED groups indicated that these four LED spectra were effective in stimulating food intake and energy homeostasis. In all four LED groups, the gonadal development advanced from chromatin nucleolar-stage oocytes to perinucleolar-stage oocytes after two months, but there were no significant differences of FSHβ and LHβ expression. This suggests that FSHβ and LHβ may not play important roles in gonadal development when fish are exposed to these four light spectra. The results of this study provide evidence that natural light conditions (white LED conditions) are a better choice for the growth of juvenile red spotted grouper in captivity during summer.

## Figures and Tables

**Table 1:** Forward and reverse primers used for quantitative real-time PCR on the target genes of red spotted grouper *(Epinephelus akaara)*.

Gene	Primer	Sequence (5′-3′)	Accession No.	Amplicon (bp)
*GH*	Forward	CGATCTCCTATCGGTTGGTGG	AY326406	128
	Reverse	CAGCAGTTCGTACGTTCGTC		
*PSS-I*	Forward	CTCCTCTCTGACCTCCTGCA	AY677120	165
	Reverse	GTTCTTGCAGCCAGCTTTCC		
*NPY*	Forward	TGCATCCTAACTTGGTGAGC	LC260519	204
	Reverse	TGGACCTCTTCCCATACCTC		
*CCK*	Forward	GACACCCACACCCTAGGAGA	LC260518	186
	Reverse	TCCGTTGACTCTGCTGTTTG		
*Kiss1*	Forward	TGCCACGACTCATTGTTGC	LC102811	225
	Reverse	AGATCCACCATCCTGACCTG		
*Kiss2*	Forward	GGCCTGATTGTTGGACAGGA	GU984383	166
	Reverse	TCTCGCTCAGGGACAAACAC		
*GPR54*	Forward	TCTCCCTGGATGGATCTTTG	MH791444	198
	Reverse	GAGCCAATCCAAATGCAGAT		
*sbGnRH*	Forward	ACTGTGTCTGCTGCTTGTGG	MF092862	192
	Reverse	TTGGCAAAAGGTGATTCCTC		
*FSHβ*	Forward	ACGTGAGACCTGCAGACGAT	KJ534537	301
	Reverse	AGTTTCTGGCCACAGGGTAG		
*LHβ*	Forward	TACAGGTCGGCAGAGTGATG	KJ534538	389
	Reverse	CTCGAAGGTGCAGTCAGATG		
*β-actin*	Forward	GAGCGTGGCTACTCCTTCAC	HQ007251	390
	Reverse	AGGAAGGAAGGCTGGAAGAG		

**Table 2:** Growth parameters (means ± SEM) of red spotted grouper *(Epinephelus akaara)* maintained under different LED spectra at the initial time, after the first month, and after the second month of the experiment.

	Light Spectra			
	White (N = 40)	Red (N = 40)	Blue (N = 40)	Green (N = 40)
Initial BW (g)	36.62 ± 0.82	38.66 ± 0.75	38.26 ± 0.81	38.97 ± 0.57
The first-month BW (g)	67.42 ± 1.76 ^a^	57.69 ± 1.52 ^b^	54.89 ± 1.61 ^bc^	50.96 ± 0.83 ^c^
Final BW (g)	72.38 ± 2.41 ^a^	69.78 ± 2.1 ^ab^	65.63 ± 1.94 ^ab^	63.2 ± 1.53 ^b^
Initial TL (cm)	15.04 ± 0.15	15.04 ± 0.1	15.51 ± 0.12	15.42 ± 0.09
The first-month TL (cm)	16.45 ± 0.13 ^a^	16.11 ± 0.13 ^ab^	15.83 ± 0.16 ^b^	15.64 ± 0.09 ^b^
Final TL (cm)	17.36 ± 0.15 ^a^	17.01 ± 0.14 ^ab^	16.82 ± 0.16 ^b^	16.77 ± 0.09 ^b^
Initial CF (g/cm^3^)	1.08 ± 0.02	1.13 ± 0.01	1.03 ± 0.01	1.07 ± 0.02
The first-month CF (g/cm^3^)	1.51 ± 0.03 ^a^	1.37 ± 0.02 ^b^	1.37 ± 0.02 ^b^	1.33 ± 0.02 ^b^
Final CF (g/cm^3^)	1.37 ± 0.02	1.4 ± 0.02	1.37 ± 0.02	1.34 ± 0.03
WGR (%)	97.65	80.50	71.54	62.18
SGR (% per day)	1.13	0.98	0.90	0.81
Initial GSI (%)	ND			
Final GSI (%)	0.05 ± 0.01	0.06 ± 0.01	0.04 ± 0.01	0.06 ± 0.01
Initial HSI (%)	1.46 ± 0.19 ^a^			
Final HSI (%)	2.41 ± 0.15 ^b^	2.04 ± 0.16 ^ab^	2.11 ± 0.24 ^ab^	1.78 ± 0.21 ^ab^

Data are shown as means ± SEM. A different letter in a row indicates a significant difference (*p* < 0.05). N, total number of experimental fish; ND, non-detectable; BW, body weight; TL, total length; CF, condition factor; WGR, weight gain rate; SGR, specific growth rate.

## Data Availability

The data that support the findings of this study are available from the corresponding author upon reasonable request.
